# Prognostic Value of Minnesota Multiphasic Personality Inventory-2 (MMPI-2) Profiles in Predicting Outcomes of Occipital Nerve Stimulation for Refractory Chronic Migraine: A Retrospective Bias-Corrected Multivariable Analysis

**DOI:** 10.1007/s11916-025-01401-3

**Published:** 2025-05-26

**Authors:** Marco Mercieri, Matteo Luigi Giuseppe Leoni, Alessia Naccarato, Omar Viswanath, Samah Said Al Sarmi, Giustino Varrassi, Marco Cascella, Roberto Arcioni

**Affiliations:** 1 Department of Medical and Surgical Sciences and Translational Medicine, Sapienza University of Rome, Rome, Italy; 2 Unit of Anesthesia, Intensive Care and Pain Management, Azienda Ospedaliera San Giovanni Addolorata, Rome, Italy; 3 Department of Anesthesiology, Creighton University School of Medicine, Phoenix, AZ USA; 4 Sultan Qaboos Comprehensive Cancer Care and Research Centre (SQCCCRC), University Medical City Muscat, Muscat, Sultanate of Oman; 5 Fondazione Paolo Procacci, Rome, 00193 Italy; 6 Department of Medicine, Surgery and Dentistry, University of Salerno, Baronissi, 84081 Italy

**Keywords:** Refractory chronic migraine, Occipital nerve stimulation, Minnesota Multiphasic Personality Inventory-2, MMPI-2, Neurostimulation outcomes, Psychological predictors

## Abstract

**Background:**

Refractory chronic migraine (rCM) is characterized by debilitating headaches that do not respond adequately to conventional medical treatments, leaving patients severely disabled. In these rare cases, central cervical spinal cord stimulation or occipital nerve stimulation (ONS) may offer a potential therapeutic option. However, these techniques are not without risks, lack clear scientific evidence, and impose a significant economic burden. Therefore, it is crucial to identify parameters that can assist physicians in selecting appropriate candidates for implantation. This study aimed to investigate the role of psychological profiles in predicting outcomes for ONS in rCM patients.

**Methods:**

We conducted a retrospective analysis on rCM patients treated with ONS at a second-level neuromodulation university facility. These patients were refractory to conventional medical treatments, including onabotulinumtoxin-A injections (monoclonal antibodies targeting CGRP were not yet available). The NRS for migraine intensity, the number of monthly migraine attacks, and drug consumption were assessed at 6-month and 12-month follow-ups post-implant. Psychological profiles were evaluated prior to OCN using the Minnesota Multiphasic Personality Inventory-2 (MMPI-2). A multivariable logistic regression model was developed to predict ONS outcomes, incorporating MMPI-2 as a covariate. The model’s accuracy and performance were assessed through non-parametric bootstrap, calibration, and discrimination analyses.

**Results:**

Twenty-three rCM patients were analysed. ONS was able to significantly reduce the pain intensity, the number of headache attacks per month, and drug consumption compared to pre-treatment levels at both 6- and 12-month follow-ups. The final multivariable logistic model at 12 months showed that the MMPI-2 Depression score was independently and negatively associated with favourable outcomes following ONS (optimism-adjusted OR 0.52, 95% CI 0.21–0.77, *p* = 0.03). The ROC curve indicated high model sensitivity (AUC: 0.96, 95% CI: 0.88–0.98), and the calibration plot revealed a good fit, with some improvement needed in mid-range predicted probabilities.

**Conclusion:**

ONS significantly reduced pain intensity, headache frequency, and drug use at the 12-month follow-up compared to pre-treatment levels. The MMPI-2 Depression score was an independent predictor of ONS failure, highlighting the importance of comprehensive psychological assessments in patient selection for ONS.

## Background

Migraine is a prevalent genetically influenced neurological disorder [1]. It has been listed by the Global Burden of Disease Survey as one of the top ten causes leading to specific disability worldwide [2]. The World Health Organization (WHO) has recognized migraine as one of the most disabling neurological disorder comparable to dementia, epilepsy, multiple sclerosis and stroke, and has suggested its inclusion in public health national plans worldwide [3]. Chronic daily headache (CDH) syndromes are a group of primary headache disorders occurring more than 15 days per month, for ≥ 4 h per day, for at least 3 months [4]. The CDH adult population has a prevalence of approximately 3–5% worldwide and includes patients with chronic tension-type headache (CTTH) and chronic migraine (CM) [5]. Compared to episodic migraine, patients with CM experience higher levels of disability, economic burden, and reduced overall quality of life [6]. Furthermore, psychiatric and medical comorbidities are frequently associated with these patients, who exhibit higher rates of major depression and suicide attempts compared to the general population [7]. Therefore, it is important to identify and treat these conditions as they may interfere with the prognosis, treatment, and the overall positive outcome of the illness [7, 8]. Several epidemiological and clinical studies have confirmed the elevated risk for mood, anxiety and sleep disorders in migraine and CDH [9–12]. Personality traits assessed by the Minnesota Multiphasic Personality Inventory-2 (MMPI-2) reveal characteristic profiles of the Hypochondriasis, Depression and Hysteria scales in CDH patients [13]. These findings are normally referred to as “neurotic profile”. Rausa et al. [14] reported that a concomitant presence of a psychiatric disorder was a dominant feature in headache patients. Psychiatric comorbidities may also be a risk factor for migraine chronicity [15]. Moreover, approximately 15% of patients with CM become refractory or intolerant to prophylactic and abortive pharmacological therapies with proven efficacy [16].

For those patients the European Headache Federation Expert Group defined specific criteria for the diagnosis of refractory chronic migraine (rCM) [17] and allowed for the consideration of surgical options when intolerance or lack of responsiveness to conservative treatments is ascertained [18].

The Neuromodulation Appropriateness Consensus Committee (NACC) of the International Neuromodulation Society recommends considering neurostimulation in rCM patients, as it represents the only procedure among all the available interventional therapies that is reversible and not destructive [19]. Large, multicenter, randomized studies and several case series have shown occipital neurostimulation (ONS) to be efficacious for the treatment of rCM [20–23]. However, the results obtained are less encouraging than hoped for, due to high heterogeneity in patient selection, surgical techniques used, and outcome evaluation [18]. A previously published study showed promising results using cervical 10-kHz spinal cord stimulation (SCS) for the treatment of rCM, but the experience is limited due to the small sample size and heterogeneous population [24]. Patient selection for ONS in rCM is crucial for achieving optimal treatment outcomes. Therefore, a comprehensive patient evaluation is essential for the successful implementation of ONS for rCM. This study aimed to evaluate whether personality profiles can aid in selecting patients for ONS. Additionally, we investigated whether it is possible to establish a cut-off score on the major clinical personality scales of the MMPI-2 questionnaire that can predict the 12-month outcomes of ONS.

## Methods

A retrospective database of rCM patients treated with ONS was implemented. The audit was conducted in a second level referral center for headache and neuromodulation at Sapienza University, Sant’Andrea Hospital, Rome, Italy, from January 2018 to March 2020. This study was approved by the Local Ethics Committee (CE100112) and performed in line with the principles of the Declaration of Helsinki. rCM was diagnosed by experienced headache specialist according to the criteria proposed by the Refractory Headache Special Interest Section of the American Headache Society [17].

Patients received onabotulinumtoxin-A injections as part of their treatment regimen, but these proved to be ineffective in managing their rCM symptoms (of note, patients included in this study were treated prior to the availability of monoclonal antibodies targeting the CGRP pathway). All rCM patients who were potential candidates for ONS underwent a psychological interview and completed the MMPI-2 questionnaire. The MMPI-2 questionnaire is a comprehensive psychological assessment tool used to evaluate personality structure and psychopathology. It is an updated version of the original MMPI and consists of 567 true-or-false statements. The MMPI-2 is designed to assess a wide range of psychological conditions and is commonly used in both clinical and research settings [25]. It also includes clinical and validity scales. The ten clinical scales measure different psychological conditions, such as depression, hysteria, paranoia, and schizophrenia. On the other hand, validity scales help determine the test-taking attitude of the respondent, ensuring that the results are accurate and not influenced by dishonesty or misunderstanding. All the considered patients completed an hourly headache diary for one month before the implant (baseline) and again at sixth month after the implant. The headache diary was designed to capture the following outcomes: numeric rating scale (NRS) for pain, on an eleven-point scale (from 0 to 10); number of headache days per month; number of headache attacks per day; and drug consumption, defined as the number of analgesic intakes. A headache day was defined as a calendar day with at least 4 h of continuous headache with an NRS > 4 (attack), or any NRS if concomitant with the intake of triptans or ergotamine [26]. Patients with medication overuse headache (MOH) were included only if their therapy had remained stable for the last 6 months. OCN was performed by two surgeons with extensive experience in neuromodulation. In all patients, we proceeded to cover the occipital region bilaterally with one or two leads, as required. Occipital nerves were firstly identified with the use of ultrasound and percutaneous leads were then inserted by ultrasound guidance in the subcutaneous tissue above the peripheral branches of the occipital nerve. Fluoroscopy was used to visualize the exact location and trajectory of the leads, ensuring optimal positioning.

### Data Analysis

Continuous variables are reported as median and interquartile range, while categorical data as relative number and percentage. A successful outcome (responder) was defined as a ≥ 30% reduction in the number of headache attacks per month, a ≥ 30% reduction in the NRS score, and a ≥ 30% reduction in drug consumption compared to pre-treatment levels at the 12-month follow-up. The Shapiro-Wilk test was used to test normality of distribution. Differences between responders and non-responders were explored using the Mann-Whitney U test, χ2 test, or Fisher’s exact test for categorical variables. Potential predictors were assessed using both univariable and multivariable logistic regression analyses to examine their potential relationship with patient outcomes. The Akaike information criterion (AIC) and the Bayesian (Schwartz) Information Criterion (BIC/SC) were used to assess the overall fit of the logistic regression models and for model selection [27]. Multicollinearity was excluded since the variance inflation factor (VIF) calculated for each covariate was < 10 [28]. Due to limited sample size of included patients, the accuracy of estimates and the performance of the model were evaluated using non-parametric bootstrap (10000 replications with replacement) [29]. The non-parametric bootstrap was chosen because it does not require any assumptions about the probability distribution of the original data set [30]. Therefore, the model was fitted on each of bootstrap samples and corrected for optimism, as previously reported [31]. ANOVA was used after the logistic regression model to account for non-linear effects. The model’s performance was evaluated using both the ROC (Receiver Operating Characteristic) curve and the confusion matrix. This study was conducted and reported following the TRIPOD (Transparent Reporting of a Multivariable Prediction Model for Individual Prognosis or Diagnosis) guidelines [32]. The Youden index method was used to determine the optimal cut-off point for the significant MMPI-2 predictor, which maximizes the difference between the true positive rate and the false positive rate across all possible cut-off values [33]. R software v4.3.2 (R Foundation for Statistical Computing, Vienna, Austria, www.r-project.org) was used for the analyses. Statistical significance was set at a two tailed p-value < 0.05.

## Results

During the study period, 105 patients with rCM, selected by the Headache Centre were referred to our clinic for ONS. Seventy-two patients (68.6%) refused to undergo neurostimulation surgery while 33 patients (31.4%) were deemed suitable for neurostimulation. Of these 33 patients, 4 patients (12%) had incomplete follow-up data, and 6 (18.2%) did not adequately complete the headache diary. Consequently, 23 patients were ultimately included in this study.

**Fig. 1:**
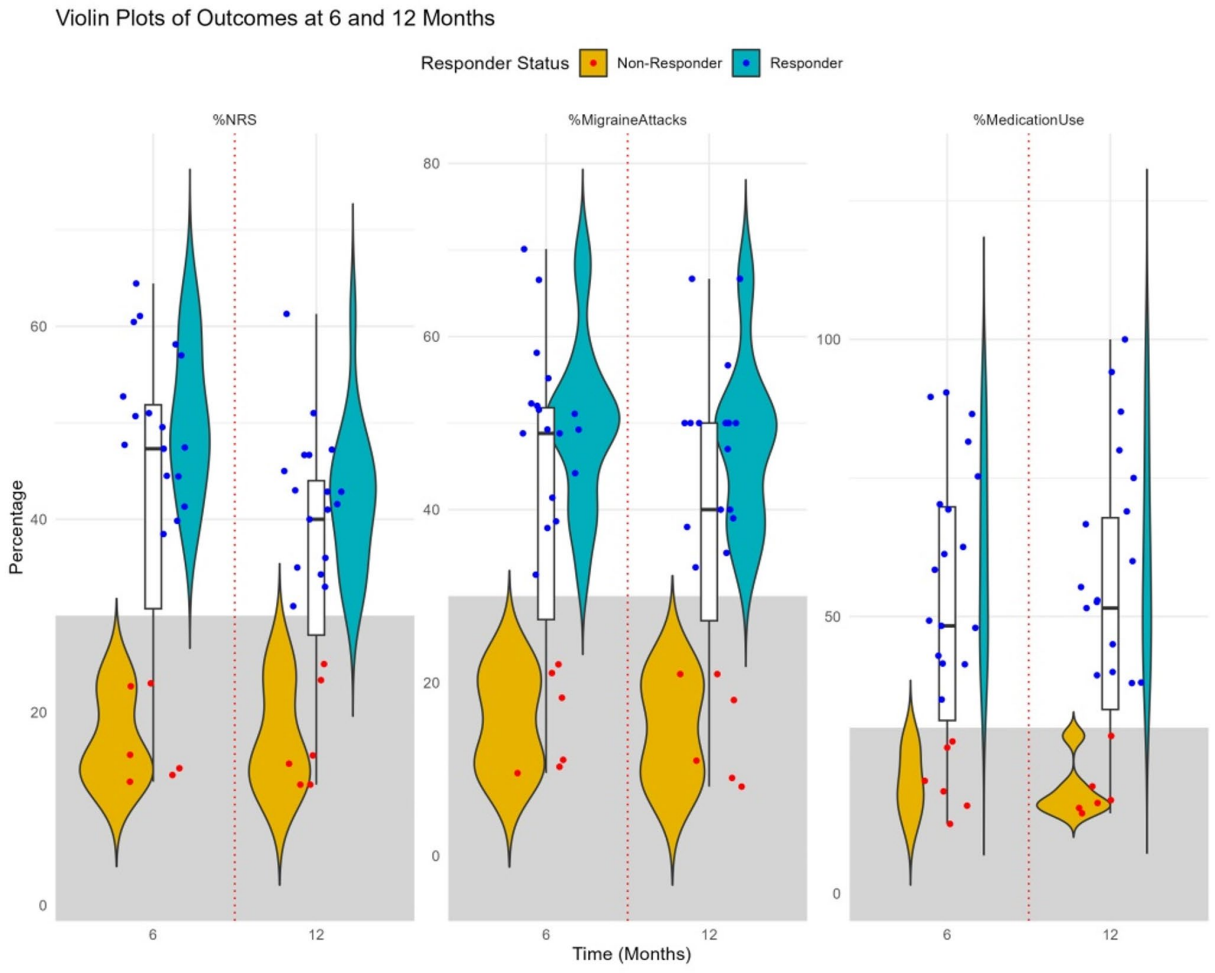
Violin plot of outcome measures in responders and non-responders. The figure shows the distribution of percentage reductions in pain intensity (NRS), number of migraine attacks, and medication use at 6 and 12 months for responders (blue) and non-responders (yellow). Each violin plot represents the data distribution, with individual patient data points jittered around the distribution. Superimposed on the violins, boxplots provide a summary of the data distribution, including the median (horizontal line within the box), the interquartile range (IQR, represented by the box). Individual data points are plotted as dots on the violins. Blue dots represent responders, while red dots represent non-responders. The grey boxes indicate the 30% threshold. Responders demonstrate significantly greater reductions in all metrics compared to non-responders

Seventeen out of 23 patients were classified as responders to OCN, showing a ≥ 30% reduction in the number of headache attacks per month, a ≥ 30% reduction in the NRS score, and a ≥ 30% reduction in drug consumption compared to pre-treatment levels at the 12-month follow-up (Fig. 1). Specifically, the NRS was reduced by 50% (44–58) in the responders at 6 months and by 43% (35–47) at 12 months, while in non-responders, it was reduced by 14% (13–23) at 6 months and 15% (13–25) at 12 months. Similarly, responders achieved greater reductions in percentage of migraine attacks (49% [41–55] at 6 months; 50% [39–67] at 12 months) and percentage of medication use (61% [48–82] at 6 months; 60% [45–75] at 12 months) compared to non-responders (percentage of migraine attacks, 15% [10–21] at 6 months; 14% [11–20] at 12 months; percentage of medication use, 20% [16–28] at 6 months; 17% [15–29] at 12 months) at both 6 and 12 months (*p* = 0.002 and *p* < 0.001, respectively).

A comparison of various demographic and psychological variables between non-responder (*n* = 6) and responder (*n* = 17) groups is reported in the Table 1. The included patients were mainly females (74%), followed by 6 males (26%), (*p* = 0.14). The median age of the responders was 54 years (IQR 49–57), compared to 51 years (IQR 45–55) in the non-responder group, *p* = 0.10. Seventeen patients (74%) achieved a good outcome after OCN at 6 months follow-up. Among the MMPI-2 scales, significant differences were observed in the Hypochondriasis (78.67 ± 13.79 in non-responders; 67.59 ± 8.40 in responders, *p* = 0.03), Depression (70.00 ± 3.58 in non-responders; 56.76 ± 7.04 in responders, *p* < 0.001), and Hysteria (79.67 ± 10.15 in non-responders; 62.35 ± 11.26 in responders, *p* = 0.003) scales. Other scales, such as Psychopathic deviate, Masculinity-femininity, Paranoia, Psychasthenia, Schizophrenia, Mania, and Social Introversion, showed no significant differences between the two groups.

Importantly, the validity scales - Lie (L), Infrequency (F), and Correction (K) - which are essential for assessing the test’s accuracy and the respondent’s approach to the questionnaire, showed no pathological results, suggesting that the questionnaire was completed accurately.

**Table 1:** Comparison of demographic and psychological variables between responder and non-responder groups

Variable	Non-responder group, *n* = 6, *n* (%)	Responder group, *n* = 17, *n* (%)	*p* value
**Age**, median (IQR), years	51 (45–55)	54 (49–57)	0.10
**Gender**			0.14
Male	0 (83%)	6 (82.5%)	
Female	6 (17%)	11 (17.5%)	
**MMPI-2 scale**			
Lie	61.50 ± 12.41	54.12 ± 10.04	0.16
F (infrequency)	53.00 ± 9.38	49.29 ± 6.24	0.29
K (correction)	54.83 ± 13.04	50.71 ± 8.76	0.39
Hypochondriasis	78.67 ± 13.79	67.59 ± 8.40	0.03
Depression	70.00 ± 3.58	56.76 ± 7.04	< 0.001
Hysteria	79.67 ± 10.15	62.35 ± 11.26	0.003
Psychopathic deviate	57.00 ± 15.61	49.71 ± 6.29	0.12
Masculinity-feminity	47.00 ± 7.32	47.71 ± 6.26	0.82
Paranoia	51.83 ± 12.81	50.35 ± 9.58	0.77
Psychasthenia	56.50 ± 12.18	51.00 ± 7.39	0.20
Schizophrenia	53.33 ± 9.11	49.53 ± 6.67	0.29
Mania	49.00 ± 5.73	47.88 ± 7.51	0.74
Social introversion	52.33 ± 7.79	51.53 ± 7.70	0.83

Based on the AIC and BIC/SC parameters of the multivariable logistic regression models, the final model included two covariates: hypochondriasis and depression. The MMPI-2 depression.score was negatively associated with good outcome after ONS (OR 0.50, 95% CI 0.19–0.75, *p* = 0.03) while hypochondriasis score was not statistically significant (OR 0.81, 95% CI 0.55–1.18, *p* = 0.26), (Table 2). After applying bootstrap correction for optimism, the analysis revealed that the depression.score remained negatively associated with favourable outcomes following ONS (optimism-adjusted OR 0.52, 95% CI 0.21–0.77, *p* = 0.03), (Table 2)

**Table 2:** Multivariable logistic regression analysis of factors associated with good outcomes after ONS, before and after bootstrap correction for optimism

Variable	OR	95% CI		*p* value	Optimism adjusted OR	Optimism adjusted 95% CI
Hypochondriasis	0.81	0.55–1.18		0.26	0.83	0.57–1.20
Depression	0.50	0.19–0.75		0.03	0.52	0.21–0.77

**Fig. 2:**
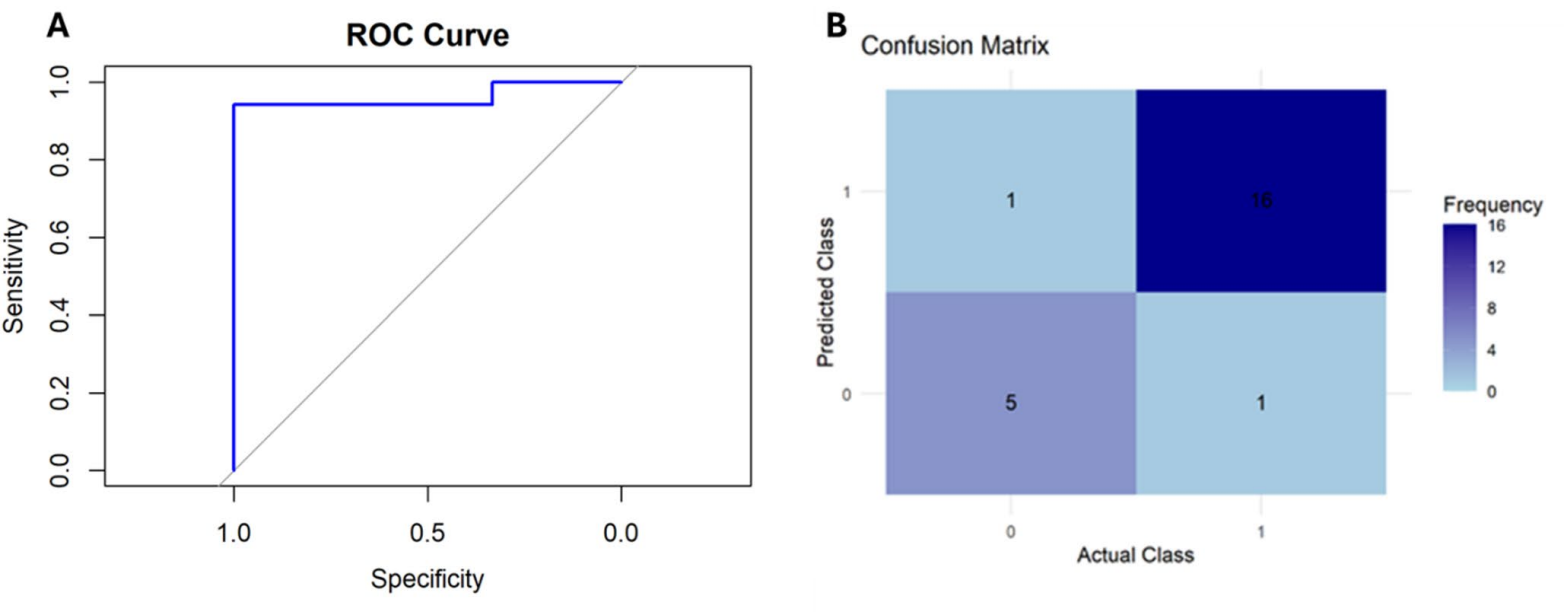
(**A**) ROC (Receiver Operating Characteristic) curve evaluating the diagnostic performance of the model. The curve plots sensitivity against 1-specificity, demonstrating the model’s ability to distinguish between positive and negative cases. (**B**) Confusion matrix illustrating the performance of the classification model. The matrix displays the frequency of predicted versus actual class labels

The ROC curve showed a sharp rise, indicating that the model achieved high sensitivity with minimal false positives and a high AUC value (AUC: 0.96, 95% CI: 0.8802-0.98), suggesting excellent model performance in effectively distinguishing between positive and negative outcomes (Fig. 2A). The confusion matrix showed 16 true positives, 5 true negatives, and only 1 false negative and 1 false positive, indicating good performance of the model in correctly predicting the actual class labels (Fig. 2B). During the study period, no complications occurred, and no explants due to loss of efficacy were recorded.

The cut-off point for the depression score, determined using the Youden index, was ≥ 63 points on the MMPI-2 questionnaire.

**Fig. 3:**
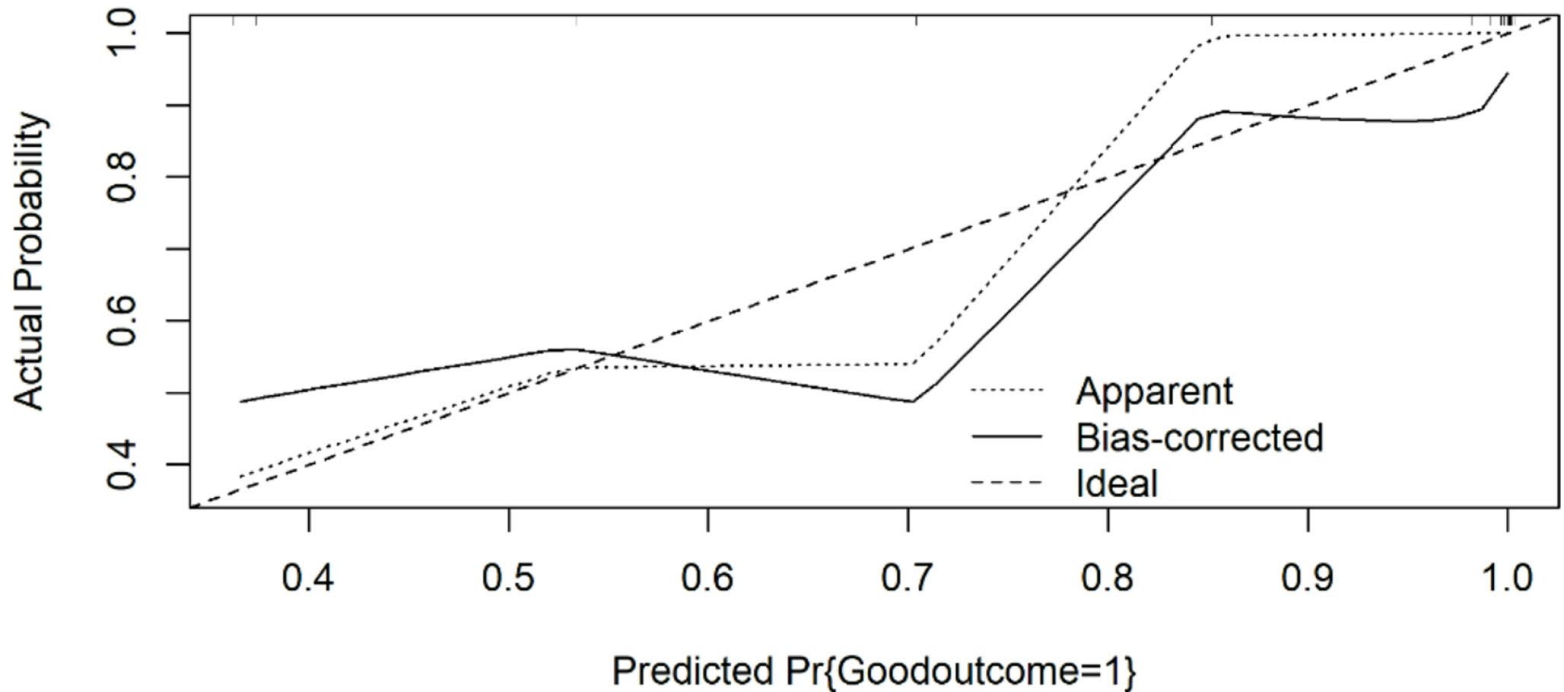
Calibration plot. The mean absolute error is 0.075, based on 10.000 bootstrap repetitions. The predictive accuracy of the model for the probability of achieving a good outcome after OCN was evaluated with a bootstrapped calibration plot. The “Apparent” line (dotted) represents the predictions directly from the model, while the “Bias-corrected” line (solid) accounts for potential overfitting by using bootstrap correction. The “Ideal” line (dashed) represents perfect calibration, where predicted probabilities match the actual outcomes exactly. For predicted probabilities < 0.6, the apparent curve is relatively close to the ideal line, indicating good initial calibration. However, for predicted probabilities between 0.6 and 0.8, there is a noticeable deviation where the model underestimates the actual probability of a good outcome. Beyond the predicted probability of 0.8, both the apparent and bias-corrected lines approach the ideal line, suggesting better calibration at higher predicted probabilities

The calibration plot after bootstrap correction revealed a mean absolute error of 0.075, indicating a reasonably good fit of the model, with some areas for improvement, particularly in the mid-range predicted probabilities (Fig. 3).

## Discussion

This study showed that ONS was effective in significantly reducing the NRS scores, the number of headache attacks per month, and drug consumption compared to pre-treatment levels at the 12-month follow-up. The MMPI-2 Depression score had a significant negative impact on predicting good outcome after ONS. Specifically, for each 1-point increase in the MMPI-2 Depression score, the odds of achieving a good outcome after ONS were reduced by 48%.

Our findings confirm that rCM involves physiological, emotional, behavioural and cognitive factors, as previously reported [26]. In fact, the physiological process of pain perception interacts with a complex interplay of these factors, along with personality traits, pain experiences, and socio-cultural influences [34]. Moreover, patients suffering from rCM frequently face stigma and adverse effects on their physical, emotional, and social well-being [35]. For these reasons, a multidisciplinary assessment and the use of headache diaries are essential steps for a correct diagnosis and to identify the most appropriate treatments.

Despite the development of various pharmacological treatments for migraines, patients often face a high risk of headache chronicity due to medication overuse [36]. To address this issue, ONS has been utilized for over 40 years as an interventional treatment for headache disorders [37]. However, previously published reports indicate that the clinical benefit varies widely, ranging from 10 to 100% at 5 months follow-up [38–41]. This variability in efficacy appears to be primarily related to differences in the surgical techniques employed over the years [42]. Progressive technological advancements in surgical techniques have led to a gradual increase in the use of ONS. However, despite these improvements in the surgical technique, the exact mechanism of action for ONS remains unknown, and various hypotheses have been suggested. First, the electrical stimulation of the occipital nerve may induce anti-nociceptive effects in the trigeminal and occipital nerves territories [43]. Furthermore, animal studies have shown that electrical stimulation of the occipital nerve can decrease the nociceptive activity of C-fibers and Aδ-fibers in the trigeminocervical complex, leading to pain relief [44]. Finally, no single mechanism predominates; instead, ONS interacts within a complex system that affects a network of structures involved in pain perception, rather than concentrating on a single structure. While the invasiveness of the procedure is reduced with the percutaneous technique, several significant challenges must be carefully considered in the clinical decision-making process. These include the possibility of adverse events for patients, such as lead migration, local immediate or delayed infections, and stimulation-related discomfort. Additionally, the collective cost of the treatment impacts healthcare resources and decision-making process [45]. All these findings highlight the importance of an accurate diagnosis, although much effort is still needed to refine patient selection for ONS.

Depression and chronic pain are intricately connected, with each condition potentially exacerbating the other. This overlap can facilitate the development of chronic pain-induced depression [46]. Moreover, depression has been identified as the single most important predictor of a SCS failure response [47]. Psychological characteristics such as catastrophizing and depression can negatively affect outcomes after spinal interventions. Interestingly, one previous study showed an improvement in depression and anxiety after SCS in patients with persistent spinal pain syndrome [48]. Moreover, the TRIUMPH study found that SCS using a passive recharge burst design was equally effective in patients with high psychological distress as in those without, suggesting that the unique waveform’s impact on the medial pain pathway may help mitigate the emotional aspects of chronic pain [49]. Although these data are encouraging, future research is needed to confirm these findings. In a retrospective study involving 83 patients with neuropathic pain, Bendinger et al. found that sleep interference is another risk factor for SCS failure [50]. In this cited study, other psychological factors, such as depression and lack of confidence in performing physical activities, also appear to be additional risk factors for a suboptimal outcome after SCS implantation. Consequently, considering the behavioural dimension will be useful in improving the predictive value to the neuromodulation. The original recommendations for selecting patients for SCS included psychological criteria like emotional stability and the absence of depression [51]. Studies show that various psychological factors, such as psychological distress, catastrophizing, history of abuse, substance use, poor social support, and cognitive deficits, are linked to poorer outcomes in pain treatment [52, 53]. Given that 50–80% of chronic pain patients exhibit signs of psychopathology [54], psychological evaluations are crucial for those being considered for interventional pain management and neurostimulation.

The MMPI-2 scale is a widely used psychological assessment tool designed to evaluate a variety of mental health conditions and personality traits. Chronic pain patients are characterized by altered MMPI-2 scores in Hypochondriasis, Depression, and Hysteria, which are the most useful discriminating factors between chronic pain patients and normal controls [55]. Elevated scores on the Minnesota Multiphasic Personality Inventory-2 -Restructured Form (MMPI-2-RF) are potential predictors of opioid misuse among chronic pain patients [56]. Similarly, the MMPI-2-RF has been useful for the early identification of somatization during the treatment of chronic pain, which helped in targeting specific treatments, such as cognitive behavioural therapy [57].

The MMPI-2 has been used to assess psychological factors in patients with migraines, aiding in the identification of underlying emotional and personality traits that may influence the frequency and severity of migraine attacks [58]. In fact, higher MMPI-2 scores have been found in migraine patients compared to healthy subjects [59]. These findings underline the crucial importance of psychological factors in patients with chronic migraine. The occurrence of depression in patients undergoing ONS, as outlined by the MMPI-2, presents a complex clinical picture. In fact, the depression may have developed after the failure of previous migraine treatments or could have been a pre-existing condition, making it challenging to determine its onset relative to the patient’s migraine journey. The presence of depression may be a risk factor for the failure of other treatments, not just ONS, as depression can negatively affect pain perception and treatment adherence, potentially undermining the efficacy of therapeutic interventions.

Although our study identified a negative predictive role for the MMPI-2 Depression score in determining a positive outcome after ONS, additional prospective studies are needed to confirm these findings in patients with rCM. Future research should focus on identifying predictive factors and objective markers for the success of ONS. Future studies should aim to evaluate and compare the relative benefits of ONS and cervical SCS in patients with rCM to determine which neuromodulation approach offers superior efficacy, safety, and patient satisfaction.

### Limitations

This study has several limitations. First, the inclusion of patients treated before the availability of CGRP monoclonal antibodies, which are known for their efficacy in migraine prevention, represents a limitation of this study, although it seems conceivable that rCM patients could share the same prognostic psychological profile even after antibodies administration. Second, it is a single-center retrospective study. However, the study was conducted at an advanced university facility with extensive experience in managing migraine patients and neuromodulation. Third, the psychological evaluation relied solely on the MMPI-2 questionnaire, without incorporating more recently developed tools like the MMPI-2-RF [60] or the newest version of the MMPI, the MMPI-3 [61]. In fact, as it was recently reported, MMPI-3 scale scores can explain up to 9% more variance in neurostimulation outcomes after accounting for pre-surgical variables [62]. Consequently, expanding the psychological evaluation with other validated instruments in future research could provide a more comprehensive assessment of psychological factors related to neurostimulation outcomes. Moreover, it is plausible to suppose that MMPI-2 scores can change over time. Consequently, our data and results capture only a specific snapshot of the complex and multifactorial psychological components associated with chronic pain. This suggests that the psychological profile of chronic pain patients is dynamic and may evolve, necessitating ongoing assessment and adaptive treatment strategies. Furthermore, it is important to recognize that depression, as identified by the MMPI-2, might have developed after previous migraine treatment failures, or it could have been present before the onset of chronic migraine symptoms. This study, therefore, cannot precisely determine when depression began in these patients. Another potential limitation is the small sample size of the included patients. This limitation is related to a high rate of patients from other regions, as they typically receive post-implant care near their local hospitals. Since our center is a second-level neuromodulation facility, many patients are often lost to follow-up after the implantation of leads. Although the model demonstrates good discriminatory capacity, the bootstrap correction revealed certain areas for improvement, particularly in the mid-range predicted probabilities. This suggests that while the model performs well overall, its predictive accuracy in mid-range scenarios could be enhanced. Addressing these deficiencies could involve incorporating additional variables that capture the nuances of mid-range probabilities more effectively. Future iterations of the model should focus on these areas to ensure robust performance across the entire spectrum of predicted probabilities. Other possible covariates, such as lifestyle habits (e.g., smoking, physical activity), and differences in pharmacological treatments, were not studied, although these elements can certainly affect the observed results. In our study, only 12-month follow-up data were collected, and no long-term follow-up data were obtained. This problem is not entirely new, as long-term outcomes in neuromodulation treatments frequently lack sufficient data [63].

## Conclusion

This study confirms that OCN is a valuable therapeutic option and is effective in significantly reducing the NRS scores, the number of headache attacks per month, and drug consumption compared to pre-treatment levels at the 12-month follow-up in well-selected rCM patients. It provides valuable insights into the role of psychological factors, particularly depression, in predicting outcomes following ONS. Our findings underscore the importance of thorough psychological assessments, to identify patients who may not benefit from ONS. Notably, the MMPI-2 Depression score emerged as a significant predictor of poor response to ONS. Despite the study’s limitations, it emphasizes the need for a multidisciplinary approach to rCM treatment and to thoroughly investigate the presence of depression, as it plays a role in predicting OCN outcome. Future research should aim to validate these findings in larger, multicenter studies and explore additional psychological and lifestyle factors that may influence ONS outcomes.

## Data Availability

Data supporting the findings of this study are available upon reasonable request from the corresponding author.

## Abbreviations

ARIMA: Auto Regressive Integrated Moving Average
GBM: Gradient Boosting Machine
RF: Random Forest
RSV: Relative Search Volume
LOESS: locally estimated scatterplot smoothing
MAE: Mean Absolute Error
MBE: Mean Bias Error
RMSE: Root Mean Square Error
R²: Coefficient of Determination
CI: Confidence Interval
PDA: Peridural Anaesthesia (German term for epidural analgesia)
UK: United Kingdom

## References

[1] Bron C, Sutherland HG, Griffiths LR. Exploring the hereditary nature of migraine. Neuropsychiatr Dis Treat. 2021;17:1183–94. 33911866 10.2147/NDT.S282562 PMC8075356

[2] James SL, Abate D, Abate KH, Abay SM, Abbafati C, Abbasi N, et al. Global, regional, and National incidence, prevalence, and years lived with disability for 354 diseases and injuries for 195 countries and territories, 1990–2017: a systematic analysis for the global burden of disease study 2017. Lancet. 2018;392:1789–858. 30496104 10.1016/S0140-6736(18)32279-7 PMC6227754

[3] Neurological Disorders. Public Health Challenges [Internet]. [cited 2024 Jul 15]. Available from: https://www.who.int/publications/i/item/9789241563369

[4] Committee HC, Olesen J, Bousser M-G, Diener H-C, Dodick D, First M, et al. New appendix criteria open for a broader concept of chronic migraine. Cephalalgia Int J Headache. 2006;26:742–6. 10.1111/j.1468-2982.2006.01172.x 16686915

[5] Ahmed F, Parthasarathy R, Khalil M. Chronic daily headaches. Ann Indian Acad Neurol. 2012;15:S40–50. 23024563 10.4103/0972-2327.100002 PMC3444216

[6] Wang S-J, Wang P-J, Fuh J-L, Peng K-P, Ng K. Comparisons of disability, quality of life, and resource use between chronic and episodic migraineurs: a clinic-based study in Taiwan. Cephalalgia Int J Headache. 2013;33:171–81. 10.1177/0333102412468668 23203506

[7] Pompili M, Di Cosimo D, Innamorati M, Lester D, Tatarelli R, Martelletti P. Psychiatric comorbidity in patients with chronic daily headache and migraine: a selective overview including personality traits and suicide risk. J Headache Pain. 2009;10:283–90. 19554418 10.1007/s10194-009-0134-2 PMC3451744

[8] Mongini F, Ibertis F, Ferla E. Personality characteristics before and after treatment of different head pain syndromes. Cephalalgia Int J Headache. 1994;14:368–73. discussion 319. 10.1046/j.1468-2982.1994.1405368.x 7828197

[9] Radat F, Swendsen J. Psychiatric comorbidity in migraine: a review. Cephalalgia Int J Headache. 2005;25:165–78. 10.1111/j.1468-2982.2004.00839.x 15689191

[10] Baskin SM, Lipchik GL, Smitherman TA. Mood and anxiety disorders in chronic headache. Headache. 2006;46(Suppl 3):S76–87. 17034402 10.1111/j.1526-4610.2006.00559.x

[11] Verri AP, Proietti Cecchini A, Galli C, Granella F, Sandrini G, Nappi G. Psychiatric comorbidity in chronic daily headache. Cephalalgia Int J Headache. 1998;18(Suppl 21):45–9. 10.1177/0333102498018s2112 9533671

[12] Juang KD, Wang SJ, Fuh JL, Lu SR, Su TP. Comorbidity of depressive and anxiety disorders in chronic daily headache and its subtypes. Headache. 2000;40:818–23. 11135026 10.1046/j.1526-4610.2000.00148.x

[13] Butcher JN, Atlis MM, Hahn J. The Minnesota multiphasic personality Inventory-2 (MMPI-2). Compr handb psychol assess vol 2 personal assess. Hoboken, NJ, US: John Wiley & Sons, Inc.; 2004. pp. 30–8.

[14] Rausa M, Cevoli S, Sancisi E, Grimaldi D, Pollutri G, Casoria M, et al. Personality traits in chronic daily headache patients with and without psychiatric comorbidity: an observational study in a tertiary care headache center. J Headache Pain. 2013;14:22. 23566048 10.1186/1129-2377-14-22 PMC3620450

[15] Buse DC, Silberstein SD, Manack AN, Papapetropoulos S, Lipton RB. Psychiatric comorbidities of episodic and chronic migraine. J Neurol. 2013;260:1960–9. 23132299 10.1007/s00415-012-6725-x

[16] Vos T, Flaxman AD, Naghavi M, Lozano R, Michaud C, Ezzati M, et al. Years lived with disability (YLDs) for 1160 sequelae of 289 diseases and injuries 1990–2010: a systematic analysis for the global burden of disease study 2010. Lancet Lond Engl. 2012;380:2163–96. 10.1016/S0140-6736(12)61729-2 PMC6350784 23245607

[17] Martelletti P, Katsarava Z, Lampl C, Magis D, Bendtsen L, Negro A, et al. Refractory chronic migraine: a consensus statement on clinical definition from the European headache federation. J Headache Pain. 2014;15:47. 25169882 10.1186/1129-2377-15-47 PMC4237793

[18] Martelletti P, Jensen RH, Antal A, Arcioni R, Brighina F, de Tommaso M, et al. Neuromodulation of chronic headaches: position statement from the European headache federation. J Headache Pain. 2013;14:86. 24144382 10.1186/1129-2377-14-86 PMC4231359

[19] Deer TR, Mekhail N, Petersen E, Krames E, Staats P, Pope J, et al. The appropriate use of neurostimulation: stimulation of the intracranial and extracranial space and head for chronic pain. Neuromodulation appropriateness consensus committee. Neuromodulation J Int Neuromodulation Soc. 2014;17:551–70. discussion 570. 10.1111/ner.12215 25112890

[20] Saper JR, Dodick DW, Silberstein SD, McCarville S, Sun M, Goadsby PJ, et al. Occipital nerve stimulation for the treatment of intractable chronic migraine headache: ONSTIM feasibility study. Cephalalgia Int J Headache. 2011;31:271–85. 10.1177/0333102410381142 PMC3057439 20861241

[21] Silberstein SD, Dodick DW, Saper J, Huh B, Slavin KV, Sharan A, et al. Safety and efficacy of peripheral nerve stimulation of the occipital nerves for the management of chronic migraine: results from a randomized, multicenter, double-blinded, controlled study. Cephalalgia Int J Headache. 2012;32:1165–79. 10.1177/0333102412462642 23034698

[22] Reed KL, Black SB, Banta CJ, Will KR. Combined occipital and supraorbital neurostimulation for the treatment of chronic migraine headaches: initial experience. Cephalalgia Int J Headache. 2010;30:260–71. 10.1111/j.1468-2982.2009.01996.x 19732075

[23] Vaisman J, Markley H, Ordia J, Deer T. The treatment of medically intractable trigeminal autonomic cephalalgia with Supraorbital/supratrochlear stimulation: a retrospective case series. Neuromodulation J Int Neuromodulation Soc. 2012;15:374–80. 10.1111/j.1525-1403.2012.00455.x 22551506

[24] Arcioni R, Palmisani S, Mercieri M, Vano V, Tigano S, Smith T, et al. Cervical 10 khz spinal cord stimulation in the management of chronic, medically refractory migraine: A prospective, open-label, exploratory study. Eur J Pain Lond Engl. 2016;20:70–8. 10.1002/ejp.692 25828556

[25] Butcher JN. Personality assessment from the nineteenth to the early twenty-first century: past achievements and contemporary challenges. Annu Rev Clin Psychol. 2010;6:1–20. 20192801 10.1146/annurev.clinpsy.121208.131420

[26] Silberstein S, Tfelt-Hansen P, Dodick DW, Limmroth V, Lipton RB, Pascual J, et al. Guidelines for controlled trials of prophylactic treatment of chronic migraine in adults. Cephalalgia Int J Headache. 2008;28:484–95. 10.1111/j.1468-2982.2008.01555.x 18294250

[27] Del Buono R, Pascarella G, Costa F, Terranova G, Leoni ML, Barbara E, et al. Predicting difficult spinal anesthesia: development of a neuraxial block assessment score. Minerva Anestesiol. 2021;87:648–54. 33325214 10.23736/S0375-9393.20.14892-2

[28] El-Dereny M, Rashwan N. Solving multicollinearity problem using ridge regression models. Int J Contemp Math Sci. 2011;6.

[29] Adjei IA, Karim R. An application of bootstrapping in logistic regression model. Open Access Libr J. 2016;3:1–9.

[30] Steyerberg EW, Harrell FE, Borsboom GJ, Eijkemans MJ, Vergouwe Y, Habbema JD. Internal validation of predictive models: efficiency of some procedures for logistic regression analysis. J Clin Epidemiol. 2001;54:774–81. 11470385 10.1016/s0895-4356(01)00341-9

[31] Leoni MLG, Moschini E, Beretta M, Zanello M, Nolli M. The modified NUTRIC score (mNUTRIC) is associated with increased 28-day mortality in critically ill COVID-19 patients: internal validation of a prediction model. Clin Nutr ESPEN. 2022;48:202–9. 35331492 10.1016/j.clnesp.2022.02.014 PMC8849901

[32] Moons KGM, Altman DG, Reitsma JB, Ioannidis JPA, Macaskill P, Steyerberg EW, et al. Transparent reporting of a multivariable prediction model for individual prognosis or diagnosis (TRIPOD): explanation and elaboration. Ann Intern Med. 2015;162:W1–73. 25560730 10.7326/M14-0698

[33] Perkins NJ, Schisterman EF. The Youden index and the optimal cut-point corrected for measurement error. Biom J Biom Z. 2005;47:428–41. 10.1002/bimj.200410133 16161802

[34] Rainville P. Brain mechanisms of pain affect and pain modulation. Curr Opin Neurobiol. 2002;12:195–204. 12015237 10.1016/s0959-4388(02)00313-6

[35] Shapiro RE, Nicholson RA, Seng EK, Buse DC, Reed ML, Zagar AJ, et al. Migraine-Related stigma and its relationship to disability, interictal burden, and quality of life: results of the OVERCOME (US) study. Neurology. 2024;102:e208074. 38232340 10.1212/WNL.0000000000208074 PMC11097761

[36] Ashina S, Terwindt GM, Steiner TJ, Lee MJ, Porreca F, Tassorelli C, et al. Medication overuse headache. Nat Rev Dis Primer. 2023;9:5. 10.1038/s41572-022-00415-0 36732518

[37] Kollenburg L, Kurt E, Mulleners W, Abd-Elsayed A, Yazdi C, Schatman ME et al. Four decades of occipital nerve stimulation for headache disorders: A systematic review. Curr Pain Headache Rep. 2024. 10.1007/s11916-024-01271-1 38907793

[38] Abhinav K, Park ND, Prakash SK, Love-Jones S, Patel NK. Novel use of narrow paddle electrodes for occipital nerve stimulation–technical note. Neuromodulation J Int Neuromodulation Soc. 2013;16:607–9. 10.1111/j.1525-1403.2012.00524.x 23106950

[39] Notaro P, Buratti E, Meroni A, Montagna MC, Rubino FG, Voltolini A. The effects of peripheral occipital nerve stimulation for the treatment of patients suffering from chronic migraine: a single center experience. Pain Physician. 2014;17:E369–374. 24850118

[40] Serra G, Marchioretto F. Occipital nerve stimulation for chronic migraine: a randomized trial. Pain Physician. 2012;15:245–53. 22622909

[41] Harland TA, Zbrzeski C, DiMarzio M, Khazen O, Staudt MD, Pilitsis JG. Craniofacial peripheral nerve stimulation: analysis of a single institution series. Neuromodulation J Int Neuromodulation Soc. 2020;23:805–11. 10.1111/ner.13145 32167229

[42] Kurt E, Kollenburg L, van Dongen R, Volkers R, Mulleners W, Vinke S. The untold story of occipital nerve stimulation in patients with cluster headache: surgical technique in relation to clinical efficacy. Neuromodulation J Int Neuromodulation Soc. 2024;27:22–35. 10.1016/j.neurom.2023.10.005 38032594

[43] Medina S, Bakar NA, O’Daly O, Miller S, Makovac E, Renton T, et al. Regional cerebral blood flow as predictor of response to occipital nerve block in cluster headache. J Headache Pain. 2021;22:91. 34384347 10.1186/s10194-021-01304-9 PMC8359299

[44] Vukovic Cvetkovic V, Jensen RH. Neurostimulation for the treatment of chronic migraine and cluster headache. Acta Neurol Scand. 2019;139:4–17. 30291633 10.1111/ane.13034

[45] Mueller O, Diener H-C, Dammann P, Rabe K, Hagel V, Sure U, et al. Occipital nerve stimulation for intractable chronic cluster headache or migraine: a critical analysis of direct treatment costs and complications. Cephalalgia Int J Headache. 2013;33:1283–91. 10.1177/0333102413493193 23814173

[46] Sheng J, Liu S, Wang Y, Cui R, Zhang X. The link between depression and chronic pain: neural mechanisms in the brain. Neural Plast. 2017;2017:9724371. 28706741 10.1155/2017/9724371 PMC5494581

[47] Sparkes E, Raphael JH, Duarte RV, LeMarchand K, Jackson C, Ashford RL. A systematic literature review of psychological characteristics as determinants of outcome for spinal cord stimulation therapy. Pain. 2010;150:284–9. 20603026 10.1016/j.pain.2010.05.001

[48] Robb LP, Cooney JM, McCrory CR. Evaluation of spinal cord stimulation on the symptoms of anxiety and depression and pain intensity in patients with failed back surgery syndrome. Ir J Med Sci. 2017;186:767–71. 28132158 10.1007/s11845-017-1565-4

[49] Hagedorn JM, Falowski SM, Blomme B, Capobianco RA, Yue JJ. Burst spinal cord stimulation can attenuate pain and its affective components in chronic pain patients with high psychological distress: results from the prospective, international TRIUMPH study. Spine J Off J North Am Spine Soc. 2022;22:379–88. 10.1016/j.spinee.2021.08.005 34419628

[50] Bendinger T, Plunkett N, Poole D, Turnbull D. Psychological factors as outcome predictors for spinal cord stimulation. Neuromodulation J Int Neuromodulation Soc. 2015;18:465–71. discussion 471. 10.1111/ner.12321 26095096

[51] Doleys DM. Psychological factors in spinal cord stimulation therapy: brief review and discussion. Neurosurg Focus. 2006;21:E1. 17341042 10.3171/foc.2006.21.6.4

[52] Evers AWM, Kraaimaat FW, van Riel PLCM, Bijlsma JWJ. Cognitive, behavioral and physiological reactivity to pain as a predictor of long-term pain in rheumatoid arthritis patients. Pain. 2001;93:139–46. 11427325 10.1016/S0304-3959(01)00303-7

[53] Jamison RN, Edwards RR, Liu X, Ross EL, Michna E, Warnick M, et al. Relationship of negative affect and outcome of an opioid therapy trial among low back pain patients. Pain Pract Off J World Inst Pain. 2013;13. 10.1111/j.1533-2500.2012.00575.x. 10.1111/j.1533-2500.2012.00575.x PMC3869183 22681407

[54] Kalso E, Edwards JE, Moore AR, McQuay HJ. Opioids in chronic non-cancer pain: systematic review of efficacy and safety. Pain. 2004;112:372–80. 15561393 10.1016/j.pain.2004.09.019

[55] Slesinger D, Archer RP, Duane W. MMPI-2 characteristics in a chronic pain population. Assessment. 2002;9:406–14. 12462761 10.1177/1073191102238153

[56] Giblin MJ, Cordaro M, Haskard-Zolnierek K, Jordan K, Bitney C, Howard K. Identifying the risk of opioid misuse in a chronic pain population: the utility of the MMPI-2-RF personality psychopathology five (PSY-5-RF) and higher-order scales. J Behav Med. 2022;45:739–49. 35913652 10.1007/s10865-022-00347-w

[57] Mickens LD, Nghiem DM, Wygant DB, Umlauf RL, Marek RJ. Validity of the somatic complaints scales of the MMPI-2-RF in an outpatient chronic pain clinic. J Clin Psychol Med Settings. 2021;28:789–97. 33619636 10.1007/s10880-021-09766-4

[58] Tan HJ, Suganthi C, Dhachayani S, Rizal AMM, Raymond AA. The coexistence of anxiety and depressive personality traits in migraine. Singap Med J. 2007;48:307–10. 17384877

[59] Albayrak GS, Saçmacı H, Albayrak L, Bozkurt G, Karaaslan Ö, İnan LE. A cross-sectional study on the personality traits of episodic and chronic migraine patients. Clin Neurol Neurosurg. 2023;227:107641. 36871391 10.1016/j.clineuro.2023.107641

[60] Sellbom M. The MMPI-2-Restructured form (MMPI-2-RF): assessment of personality and psychopathology in the Twenty-First century. Annu Rev Clin Psychol. 2019;15:149–77. 30601687 10.1146/annurev-clinpsy-050718-095701

[61] MMPI-3 [Internet]. Univ. Minn. Press. [cited 2024 Aug 2]. Available from: https://www.upress.umn.edu/test-division/mmpi-3/

[62] Marek RJ, Block AR, Ben-Porath YS. Reliability and validity of Minnesota multiphasic personality Inventory-3 (MMPI-3) scale scores among patients seeking spine surgery. Psychol Assess. 2022;34:379–89. 34855439 10.1037/pas0001096

[63] Brill S, Defrin R, Aryeh IG, Zusman AM, Benyamini Y. Short- and long-term effects of conventional spinal cord stimulation on chronic pain and health perceptions: A longitudinal controlled trial. Eur J Pain Lond Engl. 2022;26:1849–62. 10.1002/ejp.2002 PMC9543320 35761769

